# Testing Motivational Theories in Music Education: The Role of Effort and Gratitude

**DOI:** 10.3389/fnbeh.2019.00172

**Published:** 2019-07-31

**Authors:** Gloria Bernabé-Valero, José Salvador Blasco-Magraner, Carmen Moret-Tatay

**Affiliations:** ^1^Departamento de Ciencias de la Ocupación, Logopedia, Psicología Evolutiva y de la Educación, Universidad Católica de Valencia San Vicente Mártir, Valencia, Spain; ^2^Departamento de Didáctica de la Expresión Musical, Plástica y Corporal, Universidad de Valencia, Valencia, Spain; ^3^Departamento de Metodología, Psicología Básica y Psicología Social, Universidad Católica de Valencia San Vicente Mártir, Valencia, Spain

**Keywords:** motivation, effort, gratitude, musicians, Bayesian, music education

## Abstract

Acquiring musical skills requires sustained effort over long periods of time. This work aims to explore the variables involved in sustaining motivation in music students, including perceptions about one’s own skills, satisfaction with achievements, effort, the importance of music in one’s life, and perception of the sacrifice made. Two models were developed in which the variable of gratitude was included to integrate positive psychology into the motivational area of music education. The first predicts effort, while the second predicts gratitude. The models were tested using a sample of 84 music students. Both models were fitted using Bayesian analysis techniques to examine the relationship between variables and showed adequate goodness of fit. These models emphasize the role of cognition and motivation in music education and, more precisely, the relationship between effort and gratitude.

## Introduction

Musical training has recently gained increasing interest in the field of human cognition, specifically regarding its positive effects on brain development. This study aims to explore the cognitive processes involved in music education. According to Cogdill (2015), having a strong musical self-concept is a crucial component of whether students will have the motivation to persist with music. Musicians dedicate a large part of their lives to learning a diverse range of musical expertise, such as symbol comprehension, language rules, interpretation through singing or an instrument, and coordination of senses, both cognitive and physical; such extensive practice has a high associated cost. Nevertheless, those who consider music to be a fundamental part of their lives convert their musical facet into a lifestyle, profession, or passion with a high level of commitment. In many cases, the benefits of being a musician are not tangible, especially as remuneration is not always assured. However, musicians continue to invest extensive effort and time into their musical training. For this reason, cognitive processes are considered to be involved in the process of becoming a musician.

We are interested in investigating young students with different university degrees (teaching, social education, or psychology) whose scope of professional development is not exclusive to music. This would encompass those who would affirmatively answer the following questions: “Is being a musician part of your identity?”; “Do you consider yourself to be a musician?” This would allow for the fact that their main university degree may not be directly linked to music, meaning that they could be considered amateur musicians. This study focuses on the role of underlying cognitive processes, seeking to understand what variables affect musical behavior. To this end, this paper explores the cognitions of amateur musicians in terms of what they believe to have influenced their continued commitment to music. The study includes a series of cognitive variables related to global judgments on the maintenance of motivation in music students, such as perceptions of their own skills, satisfaction with achievements, effort, the importance of music in their life, and perception of the sacrifice they have made. To determine the range of variables to include in the empirical models, we drew fundamentally on: (1) the experiences of music students and their opinions on the elements that had influenced their lives in relation to musical training; and (2) a review the previous literature regarding the motivational elements underlying the processes of music education.

Music students’ opinions were obtained through a brainstorming task, in which we posed questions about what had led them to continue studying music. This procedure drew on prior studies of lay conceptions (Lambert et al., 2009; Bernabé-Valero et al., 2013). The researchers then categorized the students’ responses according to their relevance to the literature.

### Bandura Self-Efficacy

Some of the variables included in this investigation are related to Bandura’s classical theory of self-efficacy (Bandura, 1977b) – for example, musicians’ assessment of their abilities and of the effort they have devoted in their lives to musical activities; their opinions on the sacrifice music demands; and their satisfaction with the progress they have made in relation to music. This theory defines self-efficacy as “the conviction that one can successfully execute the required behavior to produce the outcomes” (Bandura, 1977b, p. 79). In perceived self-efficacy, Bandura distinguishes between the effectiveness and expectations of outcome expectations: “An outcome expectancy is defined as a person’s estimate that a given behavior will lead to certain outcomes; an expectation is the conviction that one can successfully execute the required behavior to produce the outcomes” (Bandura, 1977a, p. 193). Specifically, this approach suggests that individuals might cognitively assess their skills, particularly regarding the difficulty of the task. Several studies have emphasized the role of self-efficacy in music education (McCormick and McPherson, 2003; Ozmentes, 2008; Catalán and Orejudo, 2013), which has come to be defined as “beliefs about the capacity of one to achieve musical objectives” (Woody, 2004, p. 12). A strong association has been found between self-efficacy and performance (McPherson and McCormick, 2006), and between musical self-efficacy and self-esteem (Ozmentes, 2014). This theory of self-efficacy has been integrated into motivational models such as that described by Jones (2009): the MUSIC Model of Academic Motivation, which proposes a conceptual framework for five categories of teaching strategies: empowerment, usefulness, success, interest, and caring.

### Theory of Attribution and Theory of Expectancy-Value

Other motivational elements included in our study are the variables of music’s importance in one’s life, the effort invested, perceived musical talent, and the sacrifice devoted to music. According to the theory of attribution (Weiner, 1985), students’ attributions for or explanations of their past achievements are often important determinants of their choice, investment, and persistence in future activity. Some of this study’s previously found skill and effort to be the most common attributions given for success, while the most common attributions for failure were low capacity for learning and lack of effort. In Asmus (1986) study, 80% of music students attributed their successes and failures to internal reasons, and a large number of stable attributions for failure were reported. Austin and Vispoel (1998) also found that attributional beliefs, particularly those related to musical ability, were closely related to students’ musical self-concept and results in performance tests, and the magnitude of those relationships Such beliefs were generally higher when students reflected on past mistakes. Therefore, these authors recommend that music teachers increase their level of knowledge about students’ attributional beliefs (particularly the tendency to attribute failures to lack of ability and/or negative family influence), and encourage students to consider less stable and controllable factors (such as effort, persistence, and use of strategies and metacognition), which play an important role in determining achievement outcomes by promoting broader visions of musical development capacity (Austin and Vispoel, 1998). The theory of expectancy-value also explains why many students pursue and persist in music, while others abandon their study (O’Neill and McPherson, 2002). It posits that for students to be motivated to participate in an activity, they should value it and believe they can successfully master it in the future; expectancy is explained by the attributional variables and the value by the named variable of the “*importance that music has in the life of each person*.”

### Gratitude From the Positive Psychology

A novel variable included in this study is gratitude, measured through agreement with the statement: “I am grateful to have had the opportunity to study music.” The importance of the gratitude construct has increased in recent years, especially since the emergence of positive psychology. Although, to our knowledge, there has been no previous research linking gratitude and music, the framework of positive psychology has influenced the field of music education (Croom, 2012).

In general psychology, a large body of research has focused on gratitude (Emmons and McCullough, 2004; Watkins et al., 2004; Tsang, 2006; Froh et al., 2008; Wood, 2008; Bernabé-Valero, 2012), developing measuring instruments and models to deepen understanding of the concept. Gratitude has achieved great relevance because of its varying effects over time on psychological well-being (Wood et al., 2010; Lamas et al., 2014), spiritual well-being (Mills et al., 2015), prosocial behavior (Goei and Boster, 2005; Bartlett and DeSteno, 2006; Tsang, 2006; McCullough et al., 2008; Mikulincer and Shaver, 2010), and the maintenance of affective attachment (Algoe et al., 2008; Lambert and Fincham, 2011). However, few studies have researched gratitude within a specific context, and the predominant approach investigates gratitude at a dispositional level (Emmons and McCullough, 2004; Watkins et al., 2004).

Gratitude can be understood as a predisposition to recognizing, valuing, and responding to the positive aspects of personal existence, experienced as gifts received (Bernabé-Valero et al., 2014). The theory that underlies this definition considers the various agents toward whom it is experienced (both personal agents, including people with different degrees of relationship to the focal individual, and non-human agents, including luck, destiny, and God), as well as the object of gratitude (which may be pleasant experiences or those that generate suffering). This definition exhibits parallels with the neurocognitive approach espoused by Emmons and McNamara (2006), integrating the results found regarding gratitude with the costly signaling theory (CST; Sosis, 2003) and from the latest neuroimaging techniques. According to these authors, the process of gratitude implies that the recipient of a benefit performs the following subprocesses: (1) recognition of receiving a gift; (2) calculation of the benefits/costs associated with the gift; (3) experience of an emotion, which starts with appreciation and becomes gratitude; (4) recall of the benefits and the benefactor, as well as the beginning of the gratitude emotion, which maintains a motivational state corresponding to the benefit received.

The variable we include in this study fits with these processes and this definition in assessing whether people feel grateful for having had the opportunity to study music. More innovatively, this work explores gratitude in a particular field, as part of a specific judgment about the opportunity to study music. That is, gratitude is investigated as a specific attribution, rather than at a dispositional level (as in most prior studies. The expectancy-value theory (O’Neill and McPherson, 2002) argues that for students to be motivated to participate in an activity, they should value it and believe that they will ultimately successfully master it. The gratitude variable includes valuation of the object (the opportunity to study music, which many musicians perceive as part of their identity) and identification of the agents involved. These might be the closest and most explicit agents (such as family or educators), or the more indefinite aspects (such as the luck of having a music conservatory nearby or of passing the selection tests).

It should be noted that in the Spanish context of this study, musical education is a compulsory subject at school. However, the opportunity to continue receiving musical education after school depends on several factors, such as having: a conservatory of different levels that is proximate and accessible; financial resources to fund further training; time to attend classes; and an educational context favorable to such training. According to Fredrickson’s (1998) “broaden-and-build theory of positive emotions,” gratitude could play an important role in sustaining effort and musical development. She considers that positive emotions cause changes in cognitive activity, which can subsequently induce changes in behavior. Positive emotions also expand the possibilities of action and improve physical resources (Emmons and McCullough, 2004; Watkins et al., 2004; Tsang, 2006; Froh et al., 2008; Wood, 2008). If this positive emotions increases, it will indirectly increase that of the action, through more responses that are creative and diverse. Social resources will also be increased, since these enable the creation of social relationships, cooperation, and friendship. More precisely, gratitude, along with other positive emotions intrinsic to music, might explain the motivational component operating in musicians. For many reasons, we consider that gratitude for the opportunity to develop this facet may play an important role in their musical development, and could enrich understanding of the psychological processes involved in musicians’ development.

On the other hand, some studies have linked gratitude with other theories that are also addressed in this study, such as students’ self-efficacy. For instance, Bird (2015) found relationships between gratitude, self-efficacy, and well-being in middle-school adolescents. Jackson et al. (2014) used a structural equation model to demonstrate that generalized self-efficacy, gratitude, and hope are indicators of personal resources. They also found subjective well-being to be a latent variable, measured by self-esteem and satisfaction with life. By contrast, Rey (2010) studied how gratitude and subjective well-being are related to self-efficacy and control of learning beliefs among college students. These investigations support the integration of gratitude into motivational theories.

From the ideas generated by the musicians and the above-described arguments from scientific literature, different variables were chosen as predictors of gratitude and effort in the models to be tested. We decided to include variables related to the motivational models previously included in music education (such as perceptions about own skills, satisfaction with achievements, effort, importance of music in life, and perception of sacrifices). It was considered that outcome expectations could be evaluated from the viewpoint that music requires extensive sacrifice. The concept of sacrifice, which encompasses renunciations of other desires and interests, the activities related to music, and the expectation of self-efficacy (effectiveness expectation), was evaluated by asking the subjects how they viewed their own capacity for music. With respect to expectancy-value theory, it was considered relevant to ask about subjects’ satisfaction with their musical achievements (related to expectancy) and the importance of music in the subjects’ lives (the value attributed to music). The dependent variables we evaluated were the current effort dedicated to musical activities and subjects’ gratitude, assessing the latter by asking whether subjects were grateful to have had the opportunity to study music. These arguments motivated our selection of six variables in the models to be empirically tested: effort, importance, sacrifice, skills, satisfaction, and gratitude. The inclusion of gratitude, which has not (to our knowledge) been included in previous theoretical models, is justified by the above arguments on the relevance of introducing positive psychology elements into music education research.

Another innovative aspect of this work is our Bayesian approach. Bayesian inference is a useful statistical tool developed inartificial intelligence models, designed to examine conceived probabilistic conditional relationships between nodes. A Bayesian network is defined as a probabilistic relationship between nodes. In mathematical terms, both total probabilities are conditioned (occurrence of A given B) following Bayes’ theorem: P (A | B) = P (A ∩ B)/P (B). This study employs this technique to examine the sensitivity and dependence between variables, such as the effort involved in musical training. According to several authors (Nuzzo, 2014; Moret-Tatay et al., 2016), a complementary strategy to classical analysis might be the classical Bayesian approach: in particular, the *p*-values employed in hypothesis testing which is not used in Bayesian networks (Nuzzo, 2014). However, according to Van de Schoot et al. (2017), its popularity has grown relatively slow since 2010, as it might be considered to lack user-friendliness. Nonetheless, this analysis can be used to more precisely determine the state of a compilation of variables through observed measures, with several advantages in a multivariate analysis.

## Materials and Methods

### Participants and Measures

The study’s volunteer sample comprised 84 college musicians who had attained different levels of training (30.2% male, 69.8% female). The average age was 21.8 years (SD = 4.6), with an age range of 18–45 years. The participants were students of various university degrees (teaching, social education, and psychology) at two universities in Valencia (one public and one private). All participating students were amateur musicians (it was not their main career) and none had a degree in music. Regarding the training level attained in conservatory music studies, the distribution was as follows: 44.6% had completed study at elementary level; 39.7% at secondary level; and 10.8% at tertiary level. In other words, all participants were involved in different levels of music studies. All participants were volunteers and each participant signed the necessary informed consent documentation.

### Procedures

Different strategies were carried out: a qualitative and a quantitative approach. Firstly, we chose a series of variables associated with the previously developed Musical Profile Scale (Bernabé-Valero et al., 2016). This scale was developed by three teachers interested in the psychology of music, positive psychology, and music education, who sought to explore the relationships between the different variables involved in maintaining motivation in music students. In this qualitative approach, music students participated in a brainstorming task in which they answered posed questions about what elements were important to their musical history and what had led them to continue studying music. The answers were collected and categorized according to their semantic proximity. The authors selected opinions that occurred more frequently and had prior theoretical support, thereby obtaining a set of 10 items suitable for testing (Supplementary Appendix A). This set of items was then reformulated, converting item to a question to build the Musical Profile Scale (Bernabé-Valero et al., 2016), to which more specific questions regarding musical behavior were also added.

Secondly, we chose those variables most closely related to the theoretical models to be empirically tested. The sample was selected from two universities where the authors are based. The battery of questions was self-administered under the authors’ supervision during one of the classes, with permission from both the university and the presiding teacher.

### Materials

The variables included in this study were taken from the Musical Profile Scale (Bernabé-Valero et al., 2016) (Supplementary Appendix B). The battery of items presented to participants covered, among other matters, the level reached in regulated music studies, the hours devoted to studying music within their educational background, and their beliefs and attitudes toward music. Six items were chosen as variables for the model we tested.

Participants responded to the following items on a 7-point Likert scale, ranging from strongly disagree (1) to strongly agree (7). The six items were:

(i) Effort: “I put a lot of effort into activities related to music.”
(ii) Capacity: “I think I have a great capacity for music.”
(iii) Satisfaction: “I am pleased with the progress I have made in relation to music.”
(iv) Sacrifice: “I think that music has necessitated much sacrifice in my life.”
(v) Importance: “The importance of music in my life is…^*^ (ranging from no importance to absolute importance in my life)^*^.”
(vi) Gratitude: “I am grateful to have had the opportunity to study music.”

### Data Analysis

Our approach to data analysis focuses on using Bayesian networks. More precisely, after conducting a confirmatory factor analysis of the items employed, we first tested goodness of fit, and then tested the sensitivity of the variables.

Confirmatory factor analysis was performed using IBM SPSS 21 and AMOS 21 software. To confirm the model’s adequacy for a one factor solution, we used the absolute fit indices; the chi-square statistic χ^2^ (Joreskog et al., 1979; Saris and Stronkhorst, 1984); the comparative fit index (CFI); the normed fit index (NFI), also called delta 1; and the incremental fit index (IFI). For GFI, IFI and NFI, the values ranged between 0 and 1 and the reference value was 0.90 (Bollen, 1989; Bentler, 1990). For parsimony-adjusted indices and the root mean square error of approximation (RMSEA), the smaller its value, the better the fit, with a reference value of 0.05 (Steiger and Lind, 1980). Finally, a cluster analysis was employed (using K-Means Cluster) to try to identify structures within the data.

A descriptive model was constructed using Netica 4.2 (Norsys). We then developed a learning process to test the network. Once total and conditional probabilities in our sample were obtained, it was possible to make probabilistic inferences through Bayes’ theorem via the same software. We thereby developed our model through a tree augmented naïve (TAN) Bayes algorithm, which has previously exhibited excellent performance in its simplicity and inherent independence assumptions (Friedman et al., 1997). Sensibility was measured through analysis of the receiver operating characteristic (ROC) curve. Moreover, the goodness of fit was tested via three different indexes: logarithmic loss, quadratic loss, and spherical compensation.

First, logarithmic loss takes values between zero and infinity; values closer to zero indicate the best goodness of fit. Quadratic loss takes values from zero to two; again, values closer to zero indicate the best goodness of fit. Finally, spherical compensation takes values from zero to one; in this case, values closer to one indicate a better fit (López-Puga, 2012). Furthermore, each node was individually evaluated, in terms of sensitivity or percentage of information provided, variance of beliefs (their expected change squared), and mutual information (between nodes).

## Results

The Cronbach’s alpha value of 0.87 showed that the Musical Profile Scale has optimal internal consistency. All of the study variables were found to be positively related (see Table 1).

**TABLE 1:** Pearson coefficients for the variables under study (effort, importance, skills, satisfaction, gratitude, and sacrifice).

	**Effort**	**Importance**	**Skills**	**Satisfaction**	**Gratitude**	**Sacrifice**
Effort	1					
Importance	0.502^∗∗^	1				
Skills	0.576^∗∗^	0.468^∗∗^	1			
Satisfaction	0.692^∗∗^	0.418^∗∗^	0.504^∗∗^	1		
Gratitude	0.583^∗∗^	0.375^∗∗^	0.581^∗∗^	0.613^∗∗^	1	
Sacrifice	0.585^∗∗^	0.259^*^	0.372^∗∗^	0.629^∗∗^	0.544^∗∗^	1

^*^p < 0.05; ^∗∗^*p* < 0.001.

Differences between women and men were examined trough an independent samples *t* test. No statistically significant differences were found between these groups, although the importance variable was close (*p* = 0.054). Bartlett’s test of sphericity was *p* < 0.001 with a chi-square value of 230.13 (df = 15), and the Kaiser–Meyer–Olkin sample index value was 0.86. As expected, a single factor was confirmed, with 50.58% variance explained and an optimal goodness of fit: χ^2^/df = 1.72; CFI = 0.97; NFI = 0.93; IFI = 0.97; RMSEA = 0.09. Next, a cluster analysis was performed to obtain binary data. This was divided into high and low scores, following the approach in previous literature (Moret-Tatay et al., 2016). Two forecasting Bayesian models were constructed and evaluated, to test the effort as a target node. The first step involved creating a descriptive analysis of the data. This was necessary to establish the model’s adequacy and to examine the nodes’ sensitivity (see Table 2 for both models). In this sense, the model fit was assessed by three parameters, as described in section “Materials and Methods”: logarithmic loss, quadratic loss, and spherical compensation (López Puga et al., 2007; López-Puga, 2012).

**TABLE 2:** Stipulated percentage of each node in the Bayesian model for the effort (model 1) and gratitude (model 2) models.

	**Model 1: Effort**			**Model 2: Gratitude**		
**Nodes**	**Mutual information**	**Percentage of information**	**Variance of beliefs**	**Mutual information**	**Percentage of information**	**Variance of beliefs**
Sacrifice	0.21	22	0.06	0.06	9.86	0.01
Effort	–	–	–	0.14	22.9	0.02
Gratitude	0.15	16	0.04	–	–	–
Satisfaction	0.11	12	0.03	0.08	12.7	0.01
Skills	0.11	0.11	0.03	0.17	26.2	0.03
Importance	0.06	6.77	0.02	0.09	15.2	0.02

**FIGURE 1:**
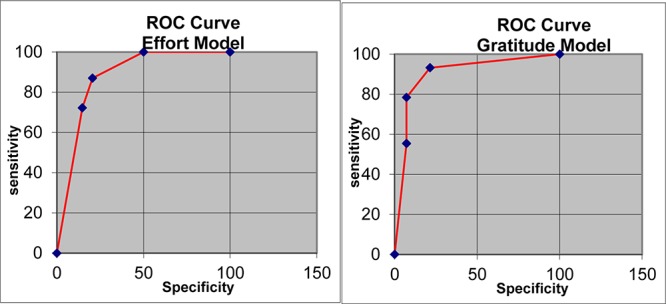
ROC (receiver operating characteristic) curve for the prediction of effort (Model 1 – left) and gratitude (Model 2 – right).

In the model to predict effort, the logarithmic loss, quadratic loss, and spherical compensation values were 0.39, 0.24, and 0.86, respectively. The area under the ROC had a value of 0.87, indicating high sensitivity (Figure 1). Next, a model to predict gratitude was tested following the same procedure. For this model, the logarithmic loss, quadratic loss, and spherical compensation values were 0.24, 0.13, and 0.93, respectively. The area under the ROC curve had a value of 0.90, again indicating high sensitivity. The skills and effort nodes had the highest percentages of information for model II.

**FIGURE 2:**
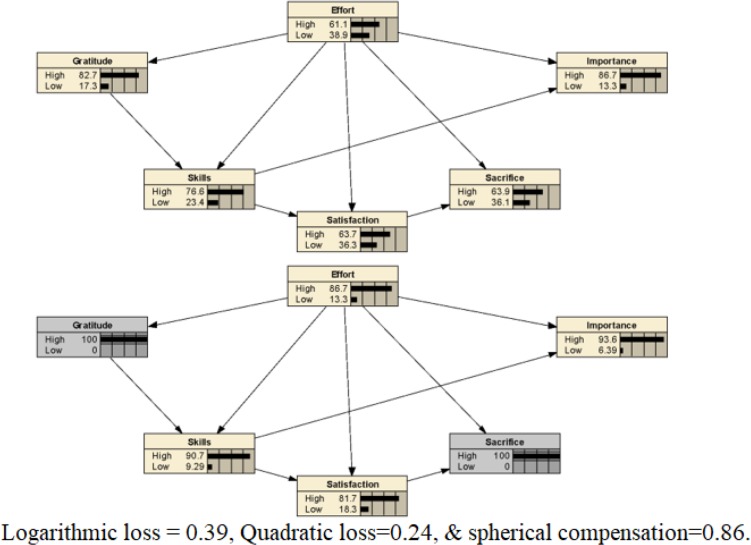
**Top:** The empirical model for predicting effort in musicians. **Bottom:** The prediction of high values in the model about effort in musicians.

**FIGURE 3:**
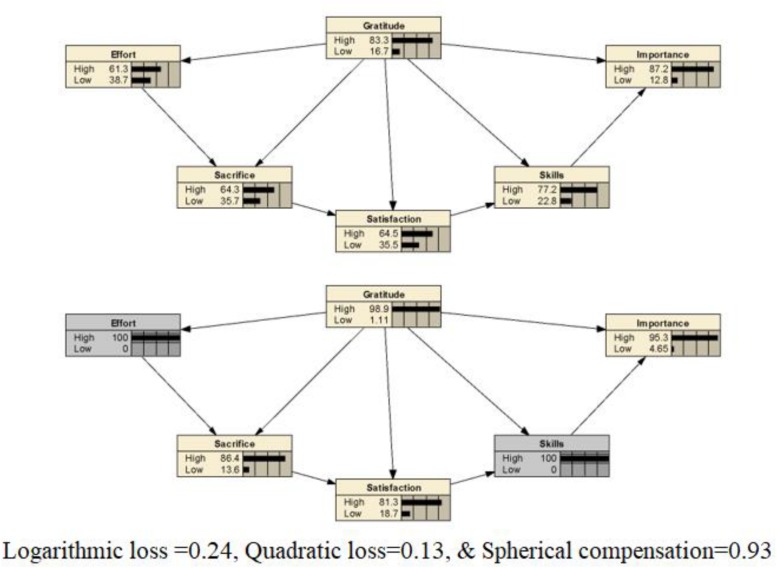
**Top:** The empirical model for predicting gratitude in musicians. **Bottom:** The prediction of high values in the model about gratitude in musicians.

Figures 2, 3 depict both the descriptive model obtained from the database (top) and *a posterior* probability distribution across the states of the true height node (bottom).

## Discussion and Conclusion

This study aimed to examine the relationship between several variables involved in the maintenance of motivation in music students, including perceptions about one’s own skills, satisfaction with achievements, effort, the importance of music in one’s life, and the perception of sacrifices made. To these variables related to the music field we added the variable of gratitude, derived from positive psychology. Our main interest was to test models of multiple relationships in which we predicted both effort on activities related to music and gratitude (for the opportunity to study music).

Another goal was to examine the relationship between the variables in terms of probabilities. Thus, we used an alternative strategy of developing a Bayesian network to examine the relationships among the variables in probabilistic terms (via Bayes’ theorem). In turn, this analysis was divided into two parts: (1) developing the model, and (2) evaluating its sensitivity. The parameters obtained in the first part of the analysis indicated that the model had adequate fit. Regarding the variables’ sensitivity, the highest percentages obtained were for “sacrifice” and “gratitude” in relation to effort (see Figure 1).

On the one hand, our model includes variables related to both efficacy-expectation and outcome-expectation (Bandura, 1977a), since the variables that consider past achievements and the self-perception of musical abilities can support the expectation of personal self-efficacy. In addition, the sacrifice variable could operate as outcome expectations as it elicited strong opinion among subjects about the large amount of practice required to achieve a good musical performance. The results of this first model have practical implications for music teachers, who could initiate metacognitive processes in their students, with the objective of forming realistic perceptions of their own skills and of the sacrifice required, as well as feelings of satisfaction with their achievements and gratitude for the opportunity to study music.

On the other hand, this first empirical model of motivation justifies the inclusion of gratitude, since this variable was found to be among the most sensitive. This result is very promising because it allows the integration of existential attitudes as motivating elements: those individuals that regard the opportunity to study music as a gift, and therefore feel grateful, will be those that dedicate the most effort to music. This element is of great importance, since gratitude is a predominantly positive emotion (Lambert et al., 2009), so its inclusion in the model supposes a greater positive emotional charge than a negative element – a quality that has sometimes been associated with the variables of effort and sacrifice. According to this model, educators wishing to promote effort in students could consider as a motivating factor for students their gratitude for the opportunity to study music, besides specific achievements. To achieve a state of motivation and satisfaction in students regarding aspects intrinsic to music, teachers could assess what each aspect brings to their lives. This reflection is especially important in the decision-making processes regarding commitment to music; thus, it would be important to emphasize not only the costs of musical activity but also the benefits.

In the second model, all the variables were found to predict gratitude for having been able to study music. However, the effort node was the most relevant for music students. Again, effort appears to have an important predictive relationship with gratitude. Effort is related to the attribution of value investigating the effect of perceptions of a task’s value (interest, usefulness, and importance) on students’ reported effort, Cole et al. (2008) found that the usefulness and importance variables significantly predicted test-taking effort and performance. This result is congruent with the results for our own model, in which the importance and satisfaction variables also fit adequately. Additionally, the skills node is relevant to gratitude: those who feel more satisfied with their abilities in music are likely also more grateful for the possibility for development that it offers, given the satisfaction assured by congruence between the task demands and the capacities put in place. Furthermore, our results seem to support previous literature, such as the models developed by Lehmann et al. (2007) that examine both intrinsic and extrinsic motivation, involving values about achieving success in a music activity, values about predicting rewarding experiences, values for the future, and evaluation of time spent or effort.

Considering everything discussed so far and the earlier findings of other authors (Bamberger, 1979; Bernabé-Valero et al., 2016), it seems that both formal and untaught dimensions play a decisive role in musical progress. This study empirically tested two motivational models, incorporating classical variables from the musical field and a novel variable originating in positive psychology. We believe that this work shows the necessity to further integrate positive existential aspects into music education research, such as flourishing (Croom, 2012), resilience (Glowinski et al., 2016), and social bonds (Freeman, 2000). As Van Der Schyff (2015) states, people may engage more deeply with musicality when they view it as a means to form richer and more compassionate relationships with their peers, communities, and the “natural” and cultural worlds they inhabit.

This work is limited by selecting the study’s sample through non-probability sampling, which might have caused distortions. In addition, the participants were amateur musicians who were studying a university degree other than music, so with the models need to be tested with professional musicians to ensure the findings are generalizable throughout the musical field. Additionally, the variables were determined inductively from the experiences of music students, and later elaborated by expert researchers. This may have introduced some bias: although the statistical analyses depicted optimal goodness of fit, they could not be generalized to the entire population of musicians. We propose that future studies should involve recruit professional musician participants through stratified and randomized sampling to assure greater generalizability of the results.

Nevertheless, we have been able to evaluate certain variables that, to our knowledge, seem underrepresented in the existing literature on musical training (such as satisfaction, sacrifice, and gratitude). It is very important for music education research to continue investigating areas that include multivariate models using various statistical methodologies (such as Bayesian networks, structural equation models, mediation models, and moderation) to enhance the robustness of findings. Reflection on these elements could be included in music teacher training programs, enabling the incorporation of these elements into their teaching and/or learning methodologies, thus favoring the purpose, projection, and meaning of the musical experience.

## Ethics Statement

All participants completed a written informed consent and the research was approved by the ethical committee at the Universidad Católica de Valencia San Vicente Mártir: UCV2017-18-28 code.

## Author Contributions

GB-V and JB-M conceived the presented idea. CM-T performed the computations and verified the analytical methods. All authors supervised the findings of this work, discussed the results, and contributed to the final manuscript.

## Conflict of Interest Statement

The authors declare that the research was conducted in the absence of any commercial or financial relationships that could be construed as a potential conflict of interest. The reviewer RT declared a shared affiliation, though no other collaboration, with one of the authors JB-M to the handling Editor.
