# Supplementary material for: Testing Motivational Theories in Music Education: The Role of Effort and Gratitude
Source: Front Behav Neurosci. 2019 Jul 31;13:172. doi: 10.3389/fnbeh.2019.00172 (PMC6689978; doi:10.3389/fnbeh.2019.00172)
Supplement: Supplementary file 1 [file Data_Sheet_1.docx]

# Appendix A

This appendix reports the most relevant and frequently occurring ideas in the brainstorming task undertaken with music students.

| Sentences chosen by agreement between judges | Reformulated items |
| --- | --- |
| Music requires a lot of sustained effort over time—physical effort and mental effort.  Music is important in life; if music is important, you invest time in it.  I have gifts / talents for music; I have capacity for music.  What I have achieved in music motivates me; I am satisfied with my achievements.  I value the opportunity I have had to study music; I feel grateful for music.  To pursue music, you have to sacrifice a lot (free time, fun, friends).  Music often tires me out; it is not an easy process.  Music ties me to my house; it requires adherence to schedules, classes, and studies, leading to a lack of freedom.  Music fulfills me; I have a purpose in life.  Music stops me getting bored; it amuses me; I do not have time to get bored. | I dedicate a great deal of effort to music-related activities.  The importance of music in my life is….  I consider that I have a great capacity for music.  I am satisfied with the achievements I have attained in relation to music.  I am grateful to have had the opportunity to study music.  I consider that music has required me to sacrifice a lot in my life.  Sometimes I have felt that life without music would be easier.  Sometimes I have felt that, without music, I would have enjoyed more freedom in life.  Sometimes I have felt that, without music, life would be emptier.  Sometimes I have felt that life without music would be more boring. |
|  |  |

# Appendix B

ESCALA DE PERFIL MUSICAL (Bernabé-Valero, et al., 2016)

1. Ser músico forma parte de mi identidad (Me considero músico/a): (SI/NO)
2. En la actualidad, dedico parte de mi tiempo a estudiar música (SI/NO)
3. En la actualidad, dedico parte de mi tiempo a tocar un instrumento (SI/NO)
4. En la actualidad asisto con regularidad a alguna agrupación musical (banda, orquesta...) (SI/NO)
5. Me dedico a la investigación en música (SI/NO)
6. Realizo creaciones musicales (SI/NO)
7. Formo parte de alguna asociación cultural dedicada a promover la música (SI/NO)
8. Escucho música frecuentemente (SI/NO)
9. Enseño música a otros (SI/NO)
10. Dependiendo de sus contestaciones en las preguntas anteriores, por favor, conteste a las preguntas entre paréntesis para añadir cualquier información al respecto que considere de interés (PREGUNTA ABIERTA)
11. Para mí la música es (marque las opciones que corresponda): (Una parte de mi identidad/Un valor elevado (intelectual, estético, espiritual, emocional, etc.) /Una afición/ Una profesión)
12. ¿A qué edad empezaste los estudios reglados de música?
13. En caso de haberlos abandonado, ¿a qué edad?
14. Grado alcanzado en los estudios de música
15. Hasta los 12 años aproximadamente, el número de horas a la semana dedicado a la música (incluyendo clases ordinarias, dedicación de estudio autónomo y asistencia a actividades musicales varias) fue de:
16. Entre los 12 años y 18 aproximadamente, el número de horas a la semana dedicado a la música (incluyendo clases ordinarias, dedicación de estudio autónomo y asistencia a actividades musicales varias) fue de:
17. A partir de los 18 años aproximadamente, el número de horas a la semana dedicado a la música (incluyendo clases ordinarias, dedicación de estudio autónomo y asistencia a actividades musicales varias) fue de:
18. En la actualidad mi dedicación de tiempo a la música es (indica aproximadamente las horas a la semana):
19. Dedico un gran esfuerzo en actividades relacionadas con la música (Escala Likert 7 puntos: Completamente en desacuerdo 1- Completamente de acuerdo 7)
20. La importancia de la música en mi vida es (Escala Likert 7 puntos: Completamente en desacuerdo 1- Completamente de acuerdo 7)
21. Considero que tengo una gran capacidad para la música (Escala Likert 7 puntos: Completamente en desacuerdo 1- Completamente de acuerdo 7)
22. Estoy satisfecho con los logros que he alcanzado en relación con la música (Escala Likert 7 puntos: Completamente en desacuerdo 1- Completamente de acuerdo 7)
23. Estoy agradecido por haber tenido la oportunidad de estudiar música (Escala Likert 7 puntos: Completamente en desacuerdo 1- Completamente de acuerdo 7)
24. Considero que la música ha requerido mucho sacrificio en mi vida (Escala Likert 7 puntos: Completamente en desacuerdo 1- Completamente de acuerdo 7)
25. En ocasiones me he planteado que la vida sin la música sería más tranquila (Escala Likert 7 puntos: Completamente en desacuerdo 1- Completamente de acuerdo 7)
26. En ocasiones me he planteado que en la vida sin la música hubiese gozado de más libertad (Escala Likert 7 puntos: Completamente en desacuerdo 1- Completamente de acuerdo 7)
27. En ocasiones me he planteado que la vida sin la música sería más vacía (Escala Likert 7 puntos: Completamente en desacuerdo 1- Completamente de acuerdo 7)
28. En ocasiones me he planteado que la vida sin la música sería más aburrida (Escala Likert 7 puntos: Completamente en desacuerdo 1- Completamente de acuerdo 7)

MUSICAL PROFILE SCALE (Bernabé-Valero et al., 2016)

1. Being a musician is part of my identity (I consider myself to be a musician) (YES / NO)

2. Currently, I dedicate part of my time to studying music (YES / NO)

3. Currently, I dedicate part of my time to playing an instrument (YES / NO)

4. At present, I regularly play in a musical group (band, orchestra ...) (YES / NO)

5. I am dedicated to music research (YES / NO)

6. I composemusical creations (YES / NO)

7. I am part of a cultural association dedicated to promoting music (YES / NO)

8. I listen to music frequently (YES / NO)

9. I teach music to others (YES / NO)

10. Depending on your answers in the previous questions, please answer the questions in parentheses to add any related information that you consider of interest (OPEN QUESTION)

11. Music is for me (check the corresponding options): (A part of my identity / A high value (intellectual, aesthetic, spiritual, emotional, etc.) / A hobby / A profession)

12. At what age did you start formal music studies?

13. If you gave them up, at what age?

14. Degree achieved in music studies

15. Up to 12 years of age, the number of hours per week devoted to music (including ordinary classes, dedication to self-study, and attendance at various musical activities) was:

16. Between the ages of 12 and 18, the number of hours per week devoted to music (including ordinary classes, dedication to self-study, and attendance at various musical activities) was:

17. From about age 18, the number of hours per week devoted to music (including ordinary classes, dedication to self-study, and attendance at various musical activities) was:

18. Currently my time dedicated to music is (indicate approximately the number of hours per week):

19. I dedicate a lot of effort to activities related to music (7-point Likert scale: 1 = Strongly disagree, 7 = Strongly agree)

20. The importance of music in my life is (7-point Likert scale:*from no importance to absolute importance in my life* )

21. I consider that I have a great capacity for music (7-point Likert scale: 1 = Strongly disagree, 7 = Strongly agree)

22. I am satisfied with my achievements in relation to music (7-point Likert scale: 1 = Strongly disagree, 7 = Strongly agree)

23. I am grateful to have had the opportunity to study music (7-point Likert scale: 1 = Strongly disagree, 7 = Strongly agree)

24. I consider that music has required me to make many sacrifices in my life (7-point Likert scale: 1 = Strongly disagree, 7 = Strongly agree)

25. I have sometimes considered that life without music would be calmer (7-point Likert scale: 1 = Strongly disagree, 7 = Strongly agree)

26. I have sometimes considered that, without music, I would have enjoyed more freedom in life (7-point Likert scale: 1 = Strongly disagree, 7 = Strongly agree)

27. I have sometimes considered that my life would be emptier without music (7-point Likert scale: 1 = Strongly disagree, 7 = Strongly agree)

28. I have sometimes considered that life without music would be more boring (7-point Likert scale: 1 = Strongly disagree, 7 = Strongly agree)
